# Zinc and Skin Disorders

**DOI:** 10.3390/nu10020199

**Published:** 2018-02-11

**Authors:** Youichi Ogawa, Manao Kinoshita, Shinji Shimada, Tatsuyoshi Kawamura

**Affiliations:** Department of Dermatology, Faculty of Medicine, University of Yamanashi, Yamanashi 409-3898, Japan; mkinoshita@yamanashi.ac.jp (M.K.); sshimada@yamanashi.ac.jp (S.S.); tkawa@yamanashi.ac.jp (T.K.)

**Keywords:** zinc, skin, acrodermatitis enteropathica, Langerhans cells, ATP, nutrition

## Abstract

The skin is the third most zinc (Zn)-abundant tissue in the body. The skin consists of the epidermis, dermis, and subcutaneous tissue, and each fraction is composed of various types of cells. Firstly, we review the physiological functions of Zn and Zn transporters in these cells. Several human disorders accompanied with skin manifestations are caused by mutations or dysregulation in Zn transporters; acrodermatitis enteropathica (Zrt-, Irt-like protein (ZIP)4 in the intestinal epithelium and possibly epidermal basal keratinocytes), the spondylocheiro dysplastic form of Ehlers-Danlos syndrome (ZIP13 in the dermal fibroblasts), transient neonatal Zn deficiency (Zn transporter (ZnT)2 in the secretory vesicles of mammary glands), and epidermodysplasia verruciformis (ZnT1 in the epidermal keratinocytes). Additionally, acquired Zn deficiency is deeply involved in the development of some diseases related to nutritional deficiencies (acquired acrodermatitis enteropathica, necrolytic migratory erythema, pellagra, and biotin deficiency), alopecia, and delayed wound healing. Therefore, it is important to associate the existence of mutations or dysregulation in Zn transporters and Zn deficiency with skin manifestations.

## 1. Introduction

In the human body, zinc (Zn) is stably maintained in the weight of 2–3 g [1]. Skin is the third most Zn-abundant tissue in the body (skeletal muscle 60%, bones 30%, liver 5%, and skin 5%) [1]. The epidermis contains more Zn compared with the dermis [2]. In the epidermis, Zn is more abundantly distributed throughout the stratum spinosum than the other three layers of keratinocytes (KCs) [3]. In the dermis, Zn concentration in the upper dermis is higher than it in the lower dermis [2]. Zn is enriched in the granules of mast cells (MCs) [4] and MCs are more abundant in the upper dermis than in the lower dermis [5,6]. Therefore, the difference in dermal MC distributions may explain the difference of Zn distributions within the dermis.

Zn is present as a divalent ion (Zn^2+^) in cells and does not need a redox reaction upon crossing the cellular membrane. Thus, the tight regulation needed to maintain Zn homeostasis in cells is assumed by two solute-linked carrier (SLC) gene families: Zn transporter (ZnT; SLC30A) and Zrt-, Irt-like protein (ZIP; SLC39A) (reviewed in [7,8,9,10]). ZnTs and ZIPs are involved in Zn efflux and uptake, respectively. 10 ZnT and 14 ZIP were identified in humans so far [7]. Besides ZnTs and ZIPs, metallothioneins (MTs) also assume Zn regulation. MTs that ubiquitously distribute in the cytoplasm contain a unique cysteine-rich amino acid sequence. This allows MTs to bind to Zn, copper, and cadmium. An excess cytosolic Zn binds to MTs, whereas Zn is released from MTs in the condition of Zn deficiency or Zn required. Consequently, MTs function as a regulator of Zn homeostasis [11].

In this review, we review the relationship between Zn and the skin, including the function and distribution of Zn, ZnTs, ZIPs, and MTs in the skin with adding new recent findings in our recent review [12] by introducing the function of Zn and Zn transporters in various types of skin cells. Additionally, we expanded the description of Zn deficiency-related human disorders accompanied with skin manifestations. We started this review by describing the physiological functions of Zn and Zn transporters in various types of cells of skin (Section 2), followed by human skin disorders caused by mutations of Zn transporters (Section 3), human skin disorders caused by dysregulation of Zn transporters (Section 4), and human skin disorders associated with Zn deficiency (Section 5).

## 2. Physiological Functions of Zn and Zn Transporters in the Skin

Zn is a cofactor for over 1000 enzymatic reactions [13,14] and is necessary for over 2000 transcription factors [15]. Zn-finger proteins function for DNA interaction, RNA packaging, activation of transcription, regulation of apoptosis, folding and assembly of protein, and lipid binding [16,17,18]. Additionally, about 10% of human proteins binds to Zn [19]. Therefore, Zn is associated with a wide variety of organic activities such as development, differentiation, and cell growth.

**Figure 1:**
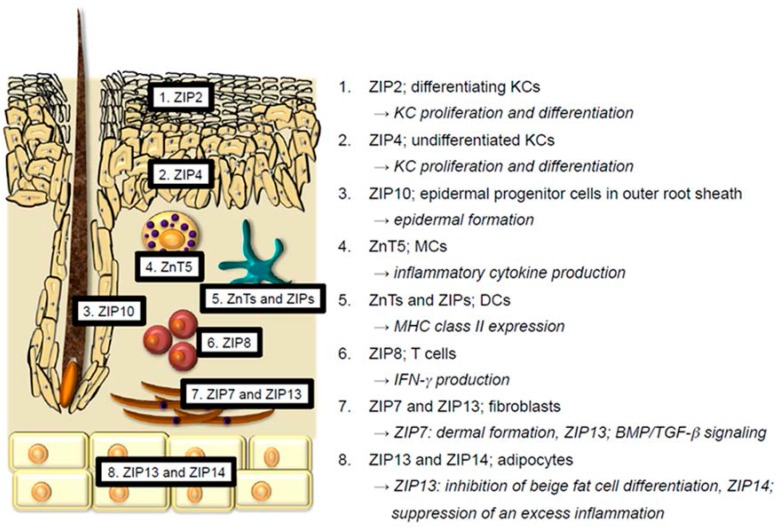
Reported distribution and function of Zn transporters in the skin. Every skin cell expresses many different Zn transporters. However, the expression and role of Zn transporters in skin cells are less understood. This figure shows the reported distribution and representative function of Zn transporters in the skin. 1 and 2. ZIP2 and ZIP4 in KCs facilitate KC proliferation and differentiation. 3. ZIP10 expressed in the epidermal progenitor cells in outer root sheath is crucial for the proper epidermal formation. 4. ZnT5 in MCs is involved in inflammatory cytokine production. 5. Many ZnTs and ZIPs regulate MHC class II expression in DCs. 6. ZIP8 in T cells is involved in interferon-γ (IFN-γ) production. 7. ZIP7 and ZIP13 in fibroblasts are required for the dermal formation and bone morphogenetic protein/transforming growth factor-β (BMP/TGF-β) signaling, respectively. 8. ZIP13 in adipocytes inhibits beige fat cell differentiation. ZIP14 in adipocytes suppresses an excess inflammation.

The skin consists of the epidermis, dermis, and subcutaneous tissue. Each skin region contains the following cells: epidermis; KCs, Langerhans cells (LCs), and melanocytes, dermis; antigen-presenting cells (dendritic cells (DCs), macrophages, and monocytes), T cells, MCs, fibroblasts, endothelial cells, etc., subcutaneous tissue; adipocytes. A review of the reported function of Zn and Zn transporters in these cells follows below (Figure 1).

### 2.1. Keratinocytes

Keratinocytes (KCs) occupy approximately 97% of epidermis and are categorized into four layers according to the degree of differentiation and keratinization. These layers include the basal layer, stratum spinosum, stratum granulosum, and stratum corneum. As described, Zn concentrations are most abundant in the stratum spinosum, more so than in the other three KC layers [3]. However, the physiological reason for this is not known. An in vitro experiment demonstrated that exposing HaCaT KCs (human immortalized KCs) to a nontoxic concentration of Zn facilitates survival and proliferation [20]. In contrast, a chelation of intracellular Zn by *N*,*N*,*N*′,*N*′-tetrakis (2-pyridylmethyl) ethylenediamine (TPEN) activates caspase-3 and DNA fragmentation, resulting in the apoptosis of KCs [21,22]. Further, a Zn-deficient diet alters the expression of keratin polypeptides in rats because of impaired keratinolytic enzyme activity [23]. In an in vitro experiment using normal human epidermal KCs, Zn suppressed IFN-γ-induced KC activation and tumor necrosis factor-α (TNF-α) production via unknown underlying mechanisms [24]. Zn also diminishes inducible nitric oxide synthase (iNOS) induction and subsequent nitric oxide (NO) production in Pam212 KCs (murine immortalized KCs) [25]. These data suggest that Zn is required for the proliferation of KCs and the suppression of inflammation in KCs (reviewed in [26]). These effects of Zn on KCs account for the clinical effects of Zn oxide ointment on skin inflammation and ulcers.

Recent research elucidated the functions of some ZIP proteins that are expressed in KCs. In mice, ZIP2 is almost exclusively expressed in the epidermis, but not in the dermis. ZIP2 is expressed in differentiating KCs. Knockdown (KD) of ZIP2 in KCs interferes with KC differentiation and proliferation. Thus, Zn uptake through ZIP2 is essential for terminal differentiation of KCs [3]. ZIP4 is associated with the development of acrodermatitis enteropathica (AE; OMIM 201100). Although ZIP4 is highly expressed in the apical side of the intestinal epithelium, human epidermal KCs also express ZIP4, particularly in undifferentitated KCs. In human epidermal KCs, ZIP4 KD reduces intracellular Zn levels up to half, interferes with normal KC differentiation, and promotes KC proliferation, thereby leading to the parakeratosis [27]. Although these histological changes by ZIP4 KD in KCs are not identical to the histological changes in AE, ZIP4, and ZIP2 are involved in the normal KC differentiation and proliferation. Zn is deeply involved in hair biology. For example, Zn deficiency (ZnD) induces telogen effluvium and abnormal hair keratinization (reviewed in [12]). Murine ZIP10 is highly expressed in the epidermal progenitor cells located in the outer root sheath of hair follicles. Thus, depletion of ZIP10 in keratin14-expressing cells results in a thin epidermis and hair follicle hypoplasia because of downregulation of the transcriptional activity of p63, a critical regulator of epidermal formation [28]. MTs are proteins that are ubiquitously expressed throughout various types of cells and predominantly distribute in the cytoplasm, and to a lesser extent in the nuclei and lysosomes [29]. MTs are comprised of four isoforms; MT-1, MT-2, MT-3, and MT-4 [30]. In the human skin, MT-1 and MT-2 are expressed in actively proliferating cells such as hair matrix, outer hair roots, and basal layer KCs [31]. These MT expressions are further upregulated in the hyperplastic KCs of inflamed skin lesions and skin cancers in conditions such as actinic keratosis, squamous cell carcinoma, and basal cell carcinoma [32]. Knock out (KO) of both MT-1 and MT-2 in mice impairs KC proliferation [33]. Thus, MT-1 and MT-2 regulate KC proliferation, likely cooperating with ZIP2 and ZIP4 in the epidermis. Similarly, MT-3 is expressed in human epidermal KCs, and its expression appears to be upregulated in skin cancers [34,35]. MT-4 expression in murine neonatal skin is reported [36]. However, the expression and function in human skin are not understood. In summary, ZIP2 and ZIP4 in KCs facilitate KC proliferation and differentiation. ZIP10 expressed in the epidermal progenitor cells in outer root sheath is crucial for the proper epidermal formation. MTs in KCs also facilitate KC proliferation.

### 2.2. Langerhans Cells

**Figure 2:**
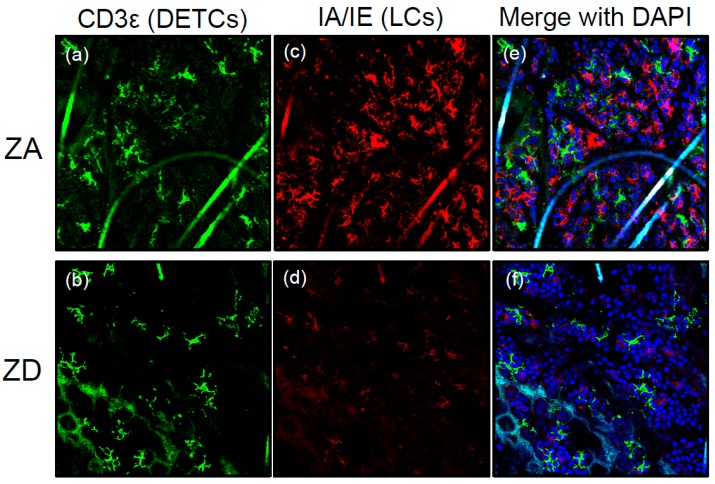
LC loss in ZD ear epidermis. Immunofluorescence of epidermal whole mounts stained for IA/IE (red) and CD3ε (green) from mice fed ZA or ZD diets for seven weeks. Original magnification, ×100. IA/IE-positive LCs are absent in ZD ear epidermis (**c**) and (**d**); whereas the distribution of CD3ε-positive dendritic epidermal T cells are unaffected (**a**) and (**b**). (**a**) and (**c**) merge with DAPI is (**e**); (**b**) and (**d**) merge with DAPI is (**f**).

Langerhans cells (LCs) are one subset of antigen-presenting cells that distribute in the epidermis [37]. LCs are involved in the development of cutaneous manifestations of acrodermatitis enteropathica (AE), a rare autosomal recessive disease. AE is caused by a mutation in the SLC39A4 gene that encodes ZIP4 followed by Zn deficiency (ZnD) because of a disability of Zn absorption within the intestines (reviewed in [38] and [39]). We found that epidermal LCs were absent in AE skin lesions. Additionally, LC loss in the epidermis was reproducible using mice with Zn-deficient diets (ZD mice) [40] (Figure 2).

**Figure 3:**
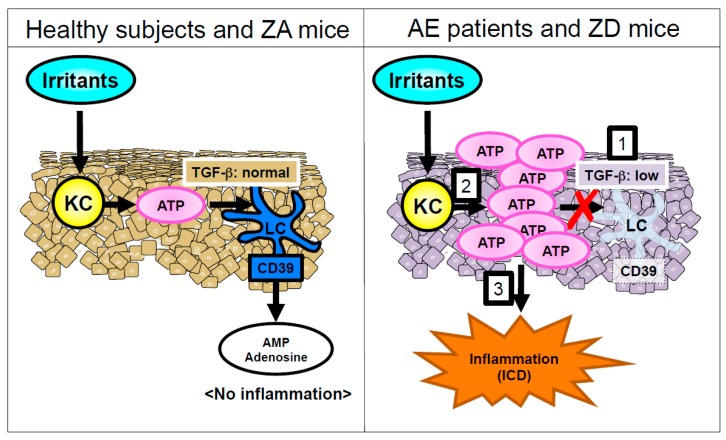
An underlying mechanism of development of acrodermatitis. Right panel: 1. In patients with AE and ZD mice, TGF-β1 expression in the epidermis is impaired compared with healthy subjects and ZA mice. Subsequently, LCs disappear from the epidermis. LCs are the sole CD39 (ecto-NTPDase1)-expressing cells in the epidermis. 2. Ear epidermis from ZD mice produces much more ATP upon the exposure of irritants than it from ZA mice. Additionally, this ATP is not hydrolyzed because of the absence of CD39-expressing LCs. 3. ATP in the epidermis elicits ICD, followed by the formation of acrodermatitis in AE.

In patients with AE and ZD mice, transforming growth factor-β1 (TGF-β1) expression in the epidermis was impaired compared with healthy people and mice with Zn-adequate (ZA) diets (ZA mice). TGF-β1 is essential for LC homeostasis [41]. Because several Zn finger transcription factors (e.g., ZNF580) are involved in TGF-β signaling [42], ZnD might decrease TGF-β1 production in LCs and KCs by influencing the activity of those transcription factors. Therefore, impaired TGF-β1 expression in the epidermis is, at least in part, responsible for LC loss (Figure 3). Another possibility for LC loss is apoptosis. In the steady state, epidermal LCs of ZD mice exhibited an increased apoptosis rate. Murine and human LCs underwent apoptosis when cultured with TPEN, whereas the culture condition did not induce apoptosis of KCs. This suggests that LCs are prone to apoptosis in the condition of ZnD. It is worth noting that LCs were replenished in the epidermis after Zn supplementation in patients with AE and ZD mice. Considered with these findings, severe ZnD induces LC apoptosis through a synergy of its direct effect and impaired TGF-β1 expression, leading to a disappearance of epidermal LC [40] (Figure 2 and Figure 3). In summary, Zn is critical for LC homeostasis. Despite the importance of Zn in LCs, the expression and function of ZnTs, ZIPs, and MTs in LCs are poorly understood.

### 2.3. Melanocytes

In an in vitro experiment, exposure of human melanocytes recovered from healthy skin to exogenous and nontoxic levels of Zn increases intracellular Zn levels, particularly in lysosomes and melanosomes. This enhances proliferation of human melanocytes with upregulated expression of AKT3, extracellular signal–regulated kinase1/2 (ERK1/2), c-MYC, and CYCD and enhances mitochondrial biosynthesis, followed by the upregulated process of autophagy [43]. In summary, Zn facilitates the melanocyte proliferation and the autophagy. The expression and function of ZnTs, ZIPs, and MTs in melanocytes are poorly understood. However, MT expression in melanoma may correlate with an increased risk of its progression and metastasis [44,45].

### 2.4. Mast Cells and Dendritic Cells

Besides the proliferative effect of Zn on various types of cells, Zn also functions as an intracellular signaling molecule, like calcium, by transducing extracellular stimuli into intracellular signaling (reviewed in [16,46]).

In mast cells (MCs), cytosolic Zn promotes FcεRI-induced granule translocation and subsequent degranulation. ZnT5 mRNA is abundant in MCs and its expression is upregulated by FcεRI stimulation. Zn and ZnT5 cooperatively translocate protein kinase C (PKC) to the plasma membrane, and the subsequent nuclear translocation of nuclear factor kappa-light-chain-enhancer of activated B cells (NF-κB), thereby promoting the production of inflammatory cytokines like IL-6 and TNF-α [47,48]. In summary, Zn and ZnT5 in MCs are involved in inflammatory cytokine production.

In dendritic cells (DCs), Zn is involved in its maturation. Upon lipopolysaccharides (LPS) stimulation of murine DCs, ZIP6 and ZIP10 are downregulated, whereas ZnT1, ZnT4, and ZnT6 are upregulated. This decreases intracellular free Zn. Because Zn promotes the endocytosis of major histocompatibility complex (MHC) class II and inhibits its trafficking from the lysosome/endosome to the plasma membrane. Decreased intracellular free Zn facilitates upregulation of surface MHC class II expression [49]. In summary, Zn and Zn transporters regulate MHC class II expression in DCs.

### 2.5. T cells

ZnD induces atrophy of the thymus accompanied by reduced double-positive thymocytes, decreasing the number of mature single positive T cells [50,51,52]. This is mediated by elevated glucocorticoids from adrenal glands and subsequent enhancement of apoptosis rate within double-positive thymocytes [53]. This suggests that Zn is required for the normal T cell generation [26].

Although the expressions and functions of ZnTs, ZIPs, and MTs in T cells are less understood, ZIP8 is enriched in human T cells, and its expression is markedly upregulated by in vitro activation with T cell receptor (TCR) engagement [54]. Although ZIP8 is localized to both the plasma membrane and lysosomes, ZIP8 in T cells is primarily localized to lysosomes. ZIP8 KD in T cells reduces IFN-γ production, whereas overexpression of ZIP8 in T cells enhances it [54], suggesting that Zn transport from lysosomes to the cytoplasm facilitates IFN-γ production in T cells. In summary, ZIP8 in T cells is involved in IFN-γ production.

### 2.6. Endothelial Cells

Zn chelation leads to death of endothelial cells (ECs). On the other hand, chronic exposure to Zn accelerates senescence of ECs in an in vitro experiment [55]. An exposure to 25 μM Zn^2+^ impedes the migration of human ECs [56]. Similarly, Zn oxide nanoparticles inhibit angiogenesis [57]. These data suggest that Zn is involved in the cell viability of ECs and angiogenesis.

Chronic exposure to Zn upregulates the mRNA expression of ZnT1 and ZIP6, downregulates the mRNA expression of ZnT5 and ZIP10, and does not alter the mRNA expression of ZIP1, ZIP2, and ZIP3 [55]. As for MTs in ECs, human ECs express MT-1E, MT-1X, MT-2A, and MT-3. An exposure to Zn induces the expression of most MT-1 isoforms. MT-2A KD leads to the decreased proliferation and the increased migration of ECs [58]. Collectively, Zn alters the expression of ZnTs, ZIPs, and MTs in ECs. However, the function of ZnTs and ZIPs in ECs remains to be identified.

### 2.7. Fibroblasts

It is known that Zn promotes lipogenesis and glucose transport via its insulin-like effects on 3T3-L1 fibroblasts and adipocytes [59]. Recent research elucidated the critical roles of ZIP7 and ZIP13 in connective tissue homeostasis. Both are intracellular Zn transporters, have quite similar amino acid sequences, and share various functional characteristics [60]. However, there are some differences. (1) ZIP7 is predominantly localized in the endoplasmic reticulum (ER), whereas ZIP13 is predominantly localized in the Golgi apparatus [60]. (2) ZIP7 is distributed throughout various types of cells, whereas ZIP13 is primarily localized in connective tissues [61,62]. ZIP7 KO mice in collagen1-expressing cells exhibit thin dermis with reduced collagen1 deposition, thin subcutaneous tissues, and a reduced number of hair follicles. Additionally, ZIP7 KD in human mesenchymal stem cells inhibits proliferation and differentiation into fibroblasts, osteoblasts, and chondrocytes [63]. As described, ZIP7 is localized in ER and transports Zn from the ER lumen to the cytoplasm. ZIP7 KD increases Zn levels in the ER, resulting in Zn-dependent aggregation and inactivation of protein disulfide isomerase, which controls proper protein folding. Thus, ER stress is overwhelming in ZIP7 KD cells, and these cells undergo apoptosis [63]. These data suggest that Zn regulation by ZIP7 in the ER is critical for the proper ER function and prevention of ER stress-induced cell death. Accordingly, the third difference between ZIP7 and ZIP13 is that ZIP7 KD induces ER stress, whereas ZIP13 KD does not induce it [64]. In conclusion, ZIP7 in fibroblasts is required for the proper dermal formation. The role of ZIP13 in fibroblasts is described later.

### 2.8. Adipocytes

ZIP13 is also involved in adipocyte biology. Adipocytes are divided into white and brown adipocytes. The former stores energy, whereas the latter consumes it. Additionally, other brown adipocyte-like cells, named beige adipocytes, are recently found within white adipose tissue, which is developed by various stimuli such as chronic cold exposure and long-term peroxisome proliferator-activated receptor γ (PPARγ) agonist treatment [65,66,67]. CCAAT-enhancer-binding protein-β (C/EBP-β) is induced in the early phases of adipogenesis and is necessary to activate PPARγ [68]. As described, ZIP13 is localized in the Golgi apparatus and transports Zn from the Golgi lumen to the cytoplasm. This Zn inhibits C/EBP-β activation and subsequent PPARγ activation, leading to the inhibition of beige fat cell differentiation. Conversely, ZIP13 KD facilitates beige fat cell differentiation. This enhances energy consumption, leading to the inhibition of obesity [69].

Inflammatory cytokines proliferate adipocytes, expanding cell mass through both hypertrophy and hyperplasia [70]. ZIP14 is localized to the plasma membrane, and its expression is upregulated by inflammatory cytokines and/or LPS [71]. Adipocytes from ZIP14 KO mice increase cytokine production by activating the NF-κB and signal transducer and activator of transcription 3 (STAT3) pathways [72]. These data suggest that ZIP14, which is upregulated in adipocytes during inflammation, may suppress excess inflammation.

Besides the involvement of ZIP13 and ZIP14 in adipocyte biology, many Zn finger proteins, which are proteins containing the Zn finger domains, are involved in adipogenesis [73].

## 3. Human Skin Disorders Caused by Mutations of Zn Transporters

Some genetic disorders are caused by mutations of Zn transporter genes. Among these disorders, mutations of ZIP4, ZIP13, and ZnT2 accompany skin manifestations.

### 3.1. ZIP4 Mutation; Acrodermatitis Enteropathica (AE; OMIM 201100)

As described in Section 2.2, AE is caused by loss-of-function mutations in ZIP4 followed by ZnD because of a disability of Zn absorption in the intestines. Since this discovery, over 30 mutations are reported [74]. The clinical symptoms are skin manifestations, alopecia, and diarrhea. Skin manifestations that are characterized by ZnD are referred to as acrodermatitis, and occur on periorificial, anogenital, and acral regions, where frequent contact with external substances is expected. Therefore, we presumed that acrodermatitis occurs as a consequence of contact dermatitis (CD). We utilized skin specimens of patients with AE and ZD mice to examine this hypothesis [40].

ZD mice exhibited an impaired allergic CD in response to dinitrofluorobenzene (DNFB) compared with ZA mice, because of an immunodeficiency in the ZD mice. On the other hand, ZD mice exhibited a significantly increased and prolonged irritant CD (ICD) in response to croton oil (CrO) compared with ZA mice. Adenosine triphosphate (ATP) is released from KCs in response to various environmental stimuli through lytic and non-lytic mechanisms [75,76,77]. ATP released from chemically injured mouse KCs causes ICD [77]. In an ex vivo organ culture, the released ATP amount from skin upon CrO application was much greater in the skin of ZD mice than in the skin of ZA mice. Additionally, an injection of apyrase that hydrolyzes ATP into adenosine monophosphate (AMP) restored the increased and prolonged ICD upon CrO application in ZD mice. These results suggest that the prolonged ICD response in ZD mice was mediated via the excess ATP release by KCs in response to irritants (Figure 3). As described in Section 2.2, epidermal LCs were absent in the lesioned skin of AE and ZD mice (Figure 2). LCs but not KCs express CD39 (ecto-nucleoside triphosphate diphosphohydrolase 1 (ecto-NTPDase1)) that potently hydrolyzes ATP into AMP [77,78]. Thus, the impaired ATP hydrolysis because of the disappearance of LCs leads to ATP-mediated inflammation in the epidermis, followed by the development of ICD (Figure 3).

### 3.2. ZIP13 Mutation; Spondylocheiro Dysplastic Form of Ehlers–Danlos Syndrome (SCD-EDS; OMIM 612350)

As described in Section 2.7, ZIP13 is localized to connective tissue cells including fibroblasts. A study with ZIP13 KO mice revealed that ZIP13 is necessary for the nuclear translocation of Smads in BMP/TGF-β signaling [61]. Thus, ZIP13 is crucial for connective tissue formation. Homozygous loss-of-function mutations of the ZIP13 gene cause SCD-EDS [61,79], characterized by hyperelastic and thin skin and hypermobility of the small joints.

### 3.3. ZnT2 Mutation; Transient Neonatal Zn Deficiency (TNZD; OMIM 608118)

Zn concentration in milk is maintained at higher levels than in serum, because Zn in breast milk is critical for the growth and survival of neonates [80]. In mice, the ZnT2 mutation causes severe ZnD in pups because of low Zn concentrations in breast milk. Like ZnT2-mutated mice, the breast milk from mice with a loss-of-function mutation of ZnT4 (lethal milk mutant mice; OMIM 602095) is deficient in Zn. Nursing pups of lethal milk mutant mice die before weaning [81]. Both ZnT2 and ZnT4 are localized in cytoplasmic secretory vesicles and efflux Zn from the cytoplasm to cytoplasmic secretory vesicles [7]. In humans, ZnT2 is essential for maintaining proper Zn concentrations in breast milk. Because breast milk from ZnT2-mutated mothers contains lower Zn, breast-feeding neonates show similar symptoms with AE [39,82,83,84,85]. On the other hand, there are no reports of ZnT4 involvement in Zn transport into secretory vesicles in humans.

## 4. Human Skin Disorders Caused by Dysregulation of Zn Transporters

Epidermodysplasia verruciformis (EV; OMIM 226400), a rare autosomal-recessive skin disease, develops non-melanoma skin cancers because of a susceptibility to oncogenic human papillomaviruses (HPVs). ZnT1 associates with the development of EV [86,87]. Although oncogenic HPVs can be detected in the skin of healthy individuals, they are asymptomatic. EV patients have mutations in either the EVER1 or EVER2 genes [88,89]. In KCs, EVER1 and 2 form a complex with ZnT1 primarily in the ER and to a lesser extent in the nuclear membrane and Golgi apparatus. The free Zn concentration in the KC nucleus is increased in patients with EV compared with healthy individuals, suggesting that the complex of ZnT1 and EVERs regulates free Zn transport in the nucleus, potentially altering cell function. A complex of ZnT1 with ‘intact’ EVERs inhibits the activator protein 1 (AP-1) activation that promotes the replication of HPV [90]. Accordingly, a complex of ZnT1 with ‘mutated’ EVERs increases free Zn transport into KC nucleus and subsequent AP-1 activity. This results in the aberrant replication of EV-related oncogenic HPVs, thereby developing skin cancers [86,87].

## 5. Human Skin Disorders Associated with Zn Deficiency

At present, acquired Zn deficiency (ZnD) still affects 17% of the world’s population who are in the condition of general malnutrition due to starvation, severe illness, alcohol addiction. Additionally, infants, the elderly, and pregnant women are also prone to fall into acquired ZnD [91,92,93,94]. Acquired ZnD could cause acquired AE. Additionally, ZnD is observed in diseases linked to nutritional deficiencies such as necrolytic migratory erythema (elevated serum glucagon), pellagra (niacin or tryptophan deficiency), and biotin deficiency. AE-like erythema is also observed in these diseases. Interestingly, loss or decrease of epidermal LCs that is seen in patients with AE is reported in some cases of diseases related to nutritional deficiencies. Loss of epidermal LCs and subsequent AE-like erythema are common phenomena in diseases related to nutritional deficiencies. Additionally, the nature of these abnormalities might be attributable to ZnD, because Zn supplementation restores these abnormalities. Besides these diseases related to nutritional deficiencies, ZnD is involved in other skin disorders.

### 5.1. Nutritional Deficiency Diseases

#### 5.1.1. Necrolytic Migratory Erythema

Necrolytic migratory erythema (NME) has been considered as a dermadrome of pancreatic glucagonoma, because NME skin lesions could resolve if the tumor is removed [95,96,97]. Patients with glucagonoma exhibit increased serum levels of glucagon. Because glucagon is involved in the metabolism of amino acids [98], excess glucagon decreases amino acids in the serum and in the epidermis, leading to epidermal necrosis.

However, it is now clarified that NME can develop within the context of other conditions including excess inflammatory mediators, liver dysfunction, and metabolic or nutritional deficiencies, particularly of Zn, and essential amino and fatty acids [97]. Because serum glucagon levels are variable in these conditions, glucagon is not the sole causative substance of NME. Decreased serum levels of Zn are reported in patients with NME suffering from inflammatory bowel disease, celiac disease, liver dysfunction, and a malignancy other than glucagonoma [99,100,101,102,103,104,105,106,107,108,109,110,111]. Supplementation of Zn restores skin lesions of NME, implying that decreased serum levels of Zn associate with the pathogenesis of NME. As described, AE-like erythema in patients with NME and its histopathological findings—such as cytoplasmic pallor, parakeratosis, subcorneal vacuolization, and ballooning degeneration of KCs—are identical to AE. In addition, one report demonstrates that epidermal LC number is reduced in patients with NME whose serum levels of Zn are decreased [111]. This suggests that similar mechanisms may underlie the development of skin lesions in patients with AE (described in Section 2.2 and Section 3.1) and NME.

#### 5.1.2. Pellagra

The compounds that have anti-pellagra activity are called niacin, and the major compound of niacin is nicotinamide and nicotinic acid. Nicotinamide is generated by two pathways. (1) Dietary nicotinic acid is promptly incorporated into the liver and is converted to nicotinamide; (2) Nicotinamide is synthesized from tryptophan, an essential amino acid, by the tryptophan–nicotinamide conversion pathway [112].

Deficiency of niacin and/or tryptophan contributes to pellagra. Diets rich in corn contain less niacin and tryptophan, contributing to the development of pellagra [113]. Patients with pellagra show AE-like erythema, diarrhea, and dementia [114]. Photosensitivity is a unique phenomenon in patients with pellagra and is not seen in other diseases associated with nutritional deficiencies. Niacin deficiency induces reactive oxygen species in KCs, followed by the production of prostaglandin E2 (PGE2). This PGE2 mediates photosensitivity in patients with pellagra [115]. A study investigating serum Zn levels in 81 pellagra patients showed a significant reduction of serum Zn level in patients with pellagra (69.7 ± 16.8 μg/dL) compared with that in healthy subjects (82.3 ± 34.0 μg/dL) [116]. Although the average serum Zn levels in patients with pellagra are in the range of latent ZnD, ZnD may be involved in the development of pellagra. Like AE and NME, loss or decrease of epidermal LCs is reported in the lesional skin of pellagra, but not in non-lesional skin of pellagra [117].

#### 5.1.3. Biotin Deficiency

Biotin is a water-soluble vitamin and serves as a co-enzyme for five carboxylases in humans. Biotin-dependent carboxylases are involved in various metabolic pathways such as gluconeogenesis, fatty acid synthesis, and amino acid synthesis. Mammals cannot synthesize biotin. However, because biotin is contained in a wide range of foods and some gut microbiota produce biotin, biotin deficiency (BnD) does not occur in people who consume a mixed general diet. On the other hand, genetic deficiency of holocarboxylase synthetase and biotinidase; continuous consumption of raw egg whites; parenteral nutrition; and modified milk without biotin supplementation can lead to the development of BnD [118]. BnD causes similar symptoms as AE including skin lesions, alopecia, and diarrhea [119]. As for the underlying mechanism of development of the skin lesions in BnD, BnD causes abnormalities in fatty acid composition such as accumulation of odd-chain fatty acids and abnormal metabolism of long-chain polyunsaturated fatty acids [120,121,122,123]. Besides fatty acid abnormalities, ZnD is reported in some patients with BnD [124,125,126]. Although the serum Zn levels in patients with BnD are inconsistent among reports [127,128,129], ZnD might contribute to the development of BnD when considering their similar symptom profiles.

### 5.2. Alopecia

Alopecia is roughly classified into non-scarring and scarring. The former includes telogen effluvium, alopecia areata, and androgenic alopecia [130]. ZnD is related to some non-scarring alopecia.

#### 5.2.1. Alopecia in Acrodermatitis Enteropathica

Alopecia developed in patients with acrodermatitis enteropathica (AE) shows the characteristics of telogen effluvium (TE), which is a type of non-scarring alopecia and is defined by the premature transition of anagen to telogen phase [131,132]. Patients with TE without AE also exhibit decreased serum levels of Zn compared with healthy individuals [132,133]. TE can be restored by supplementation of Zn [132]. In patients with AE, hair contains less Zn [134] and hair shafts show a characteristic irregular pattern because of impaired incorporation of cystine, a major content of hair keratin amino acids that is required for normal hair keratinization [135,136]. This is evidenced by impaired incorporation of radiolabeled cystine into hair in ZD rats [137,138,139]. Although alopecia-like hair loss is reported in dietary ZD AE models using mice [140], rats [141,142], and rabbits [143], no studies have addressed the underlying mechanisms. In summary, ZnD induces TE and abnormal hair keratinization.

#### 5.2.2. Alopecia Areata

Alopecia areata (AA) is an autoimmune disease mediated by cytotoxic T lymphocytes [144]. IFN-γ KO mice do not develop experimental AA [145,146]. The association between AA and lower serum levels of Zn is a subject of active inquiry. The results of multiple analyses of serum Zn levels and AA are contradictory (reviewed in [12]). However, evidence indicates that serum levels of Zn are certainly decreased in patients with severe AA whose alopecia is broad and prolonged and is resistant to conventional therapies [147,148,149]. This implies that the serum levels of Zn are a useful parameter to predict the severity and that supplementation of Zn can be a promising adjuvant therapy, along with standard therapy for severe AA.

### 5.3. Cutaneous Wounds and Ulcers

The process of wound healing is complex and involves various Zn-related molecules, such as MTs, matrix metalloproteinases, integrins, alkaline phosphatase, and Zn finger proteins [150,151]. Oral and/or topical Zn has been long used to treat ulcers and wounds. Unexpectedly, a systematic review using data from the Cochrane Wound Group failed to conclude that oral Zn supplementation improves wound healing [152]. However, studies show the efficacy of topical Zn oxide for improving rates of wound healing, regardless of serum Zn levels of patients [153,154].

As described in Section 2.4, Zn acts as an intracellular signaling molecule. G-protein coupled receptor 39 (GPR39) has been identified as a Zn-sensing receptor [155]. GPR39 is an orphan G protein-coupled receptor that is conserved in vertebrates and transduces autocrine and paracrine Zn signals [155]. In an in vitro experiment, Zn released from injured KCs stimulates GPR39, and this signaling promotes epithelial repair [156]. Stem cells in the skin are localized in various regions including the interfollicular epidermis, hair follicles, sweat glands, and sebaceous glands (SGs) [157]. These stem cells are critical for wound repair [157]. In murine skin, GPR39 is exclusively localized to SGs and is co-localized with stem cells in SGs that express Blimp1. GPR39 KO mice show delayed wound healing [158]. These data demonstrate that Zn signaling through GPR39 plays an important role in wound healing by stimulating stem cell activity in SGs.

### 5.4. Other Skin Disorders Associated with Zn Deficiency

Zn deficiency (ZnD) is reported in some skin disorders including inflammatory diseases (atopic dermatitis [159,160], oral lichen planus [161], and Behcet’s disease [162,163]), autoimmune bullous diseases (pemphigus vulgaris [164] and bullous pemphigoid [165]), inherited bullous diseases (inherited epidermolysis bullosa [166,167]), and hyperpigmentation (melasma [168]). Because Zn is deeply involved in the regulation of immune systems, it is likely that ZnD leads to the development of these inflammatory and autoimmune disorders [26,169].

Accumulation of evidence is required to determine the relationship between Zn and these skin disorders.

## 6. Conclusions

The association of skin manifestations and ZnD is well known. AE is caused by the mutations of ZIP4 and subsequent ZnD. Substantial numbers of patients suffering from diseases related to nutritional deficiencies such as acquired AE, NME, pellagra, and BnD show low serum Zn levels. This suggests that ZnD affects any cell type. Additionally, many micronutrients are also deficient in the conditions that fall into nutritional ZnD. Patients with these disorders share common skin manifestations of acrodermatitis. Additionally, LC loss in the epidermis is reported in patients with AE, NME, and pellagra. LCs are the sole CD39 (ecto-NTPDase1)-expressing cells in the epidermis. Thus, the disappearance of LCs in the epidermis exacerbates skin inflammation after irritant exposure (Figure 2 and Figure 3). In conclusion, acrodermatitis in diseases associated with nutritional deficiencies may be caused by ICD, secondary to LC loss.

Although every cell expresses many different Zn transporters, which all contribute to homeostatic control of the cells and tissues, the role of Zn transporters in skin homeostasis is less understood. However, recent studies add some important information to this field (Figure 1). For instance, ZIP2 and ZIP4 contribute to KC proliferation and differentiation. ZIP10 is critical for skin homeostasis and epidermal formation. ZIP7 is important for proper dermal formation. ZIP13 is involved in adipocyte biology.

ZnD is a current problem in both developing and developed countries. We have to pay attention to cutaneous symptoms in order not to miss the ‘dermadrome’ of ZnD.
